# The Role of Social Mobilization in Controlling Ebola Virus in Lofa County, Liberia

**DOI:** 10.1371/currents.outbreaks.c3576278c66b22ab54a25e122fcdbec1

**Published:** 2015-05-15

**Authors:** Shannon M. Fast, Sumiko Mekaru, John S. Brownstein, Timothy A. Postlethwaite, Natasha Markuzon

**Affiliations:** Software and Algorithms, Draper Laboratory, Cambridge, Massachusetts, USA; Boston Children's Hospital, Emergency Medicine, Boston, Massachusetts, USA; Boston Children’s Hospital, Harvard Medical School, Boston, Massachusetts, USA; Biomedical Systems, Draper Laboratory, Cambridge, Massachusetts, USA; Information and Decision Systems, Draper Laboratory, Cambridge, Massachusetts, USA

**Keywords:** disease model, ebola, human behavior, social mobilization

## Abstract

The West Africa Ebola virus epidemic now appears to be coming to an end. In the proposed model, we simulate changes in population behavior that help to explain the observed transmission dynamics. We introduce an EVD transmission model accompanied by a model of social mobilization. The model was fit to Lofa County, Liberia through October 2014, using weekly counts of new cases reported by the US CDC. In simulation studies, we analyze the dynamics of the disease transmission with and without population behavior change, given the availability of beds in Ebola treatment units (ETUs) estimated from observed data. Only the model scenario that included individuals' behavioral change achieved a good fit to the observed case counts. Although the capacity of the Lofa County ETUs greatly increased in mid-August, our simulations show that the expansion was insufficient to alone control the outbreak. Modeling the entire outbreak without considering behavior change fit the data poorly, and extrapolating from early data without taking behavioral changes into account led to a prediction of exponential outbreak growth, contrary to the observed decline.  Education and awareness-induced behavior change in the population was instrumental in curtailing the Ebola outbreak in Lofa County and is likely playing an important role in stopping the West Africa epidemic altogether.

## Introduction

It has long been recognized that human behavior can have a dramatic influence on the spread of infectious disease 1 ^,^ 2. The joint dynamics of disease transmission and social processes often differ substantially from the dynamics of either operating in isolation 3, and mathematical models of disease transmission are beginning to incorporate the effects of behavior change 3 ^,^ 4 ^,^ 5. The effect of human behavior is particularly pronounced for Ebola virus disease (EVD) outbreaks, with extreme responses observed in prior outbreaks 6 ^,^ 7.

The 2014 West Africa EVD epidemic is the largest ever recorded, with 25,826 cases and 10,704 deaths reported by April 12, 2015 8, primarily in Guinea, Liberia and Sierra Leone. Ebola virus outbreaks are brought under control by rapidly isolating symptomatic individuals, tracing their contacts, safely burying or cremating fatalities 9, and changing population behavior toward better protective practices. These infection control measures require not only increased medical staff and hospital capacity but also the support and cooperation of the population 10 ^,^ 11. Social mobilization efforts include media campaigns, door-to-door outreach and education programs within churches and mosques 10. With limited resources available to control the epidemic, it is important to understand which combination of measures is most effective. Several studies suggest that increasing the capacity of Ebola Treatment Units (ETUs) is instrumental in bringing EVD outbreaks under control 12 ^,^ 13 ^,^ 14 ^,^ 15. We present a network-based model of EVD transmission, in which we combine isolation of cases in ETUs with a change in the public's cooperation with preventative measures to control EVD spread. This model builds upon previous models that merge disease transmission and social processes 16 ^,^ 17 ^,^ 18 ^,^ 19, including several models that address social processes affecting emerging infectious disease transmission 20 ^,^ 21 ^,^ 22. While many EVD models have addressed behavior change and other time-varying properties of the disease dynamics **** 12 ^,^ 13 ^,^ 14 ^,^ 15 ^,^ 23 ^,^ 24, this model is among the first to explicitly account for the change in attitudes that underlies behavior change. Importantly, we differentiate between delay in hospitalization resulting from lack of ETU capacity and delay resulting from reluctance to pursue medical treatment. As a result, we can isolate the effect of social mobilization efforts aimed at increasing treatment-seeking behavior.

We fit the model to weekly case counts from Lofa County, Liberia, in order to analyze the importance of both social mobilization efforts and the expansion of ETUs in containing the spread of EVD. Lofa County is located in northern Liberia and is a largely rural area, historically known as Liberia's bread basket 25. Although an early Lofa County EVD outbreak was controlled, an imported case launched a much larger outbreak in late-May 26, which infected 623 people 27. The response consisted of expansion of treatment and testing facilities and a social mobilization campaign. Though the response was initially met with resistance and even violence 28 ^,^ 29 ^,^ 30, social mobilization efforts proved successful at later stages 31 ^,^ 32, and led to a greater cooperation with control measures. Cases were more rapidly isolated, unsafe burial practices were stopped, and the number of new cases per week began to decline (Figure 1)

**Newly reported cases of EVD by week in Lofa County, Liberia according to the Liberian Ministry of Health and Social Welfare and local health offices (CDC compiled weekly case counts).**
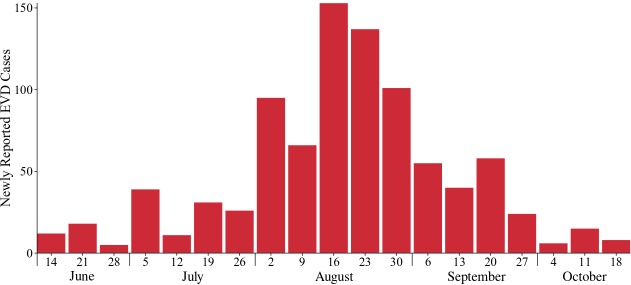
The outbreak began in late-May and worsened through mid-August, when the number of new cases per week began to decline. Due to reclassification of suspected and probable cases, the reported cases per week do not always correspond with the reported cumulative cases.

By modeling the spread of the disease and analyzing the effect of changes in population compliance with control measures, we show that the observed ETU capacity in Lofa County would have been insufficient to stop the disease outbreak had population behavior not changed. Our simulations suggest that behavioral change is a key reason for the slowing of the Lofa County outbreak. We discuss how this conclusion can affect policy for EVD containment.

## Methods

We implement an EVD transmission model 33on a contact network, in which individuals are represented as nodes and disease-spreading contacts between them are represented as edges. The model simulates the status of each individual in the population on each day t. The stages of EVD represented in the model are summarized in Figure 2. Our model of EVD transmission is overlaid with a model of social mobilization, leading to increased awareness of the disease. As a result of increased awareness, we assume that individuals take steps to prevent further transmission, as described in later sections.

**State transitions for individuals in the EVD transmission model.**
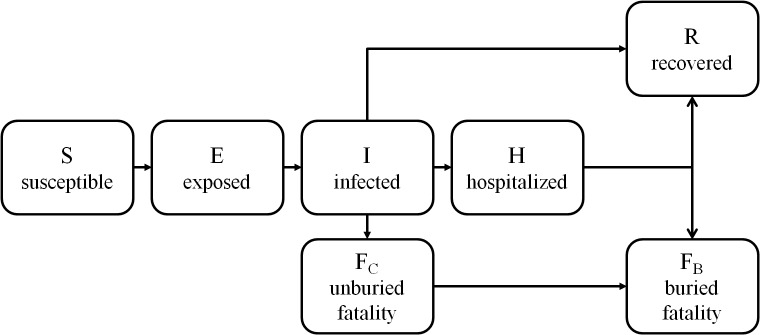

**EVD Transmission and Case Isolation Model**

The EVD transmission model is an extension of the standard susceptible-infected-recovered model 34. Each individual i's disease state at time t is represented by X_i_(t) ∈ {S, E, I, H, F_C_, F_B_, R} where:

**Table:** 

State	Description
S	susceptible to EVD infection
E	exposed to EVD but not yet infectious
I	infectious and in the community
H	infectious and isolated in an ETU
F_C_	fatality that is unburied and in the community
F_B_	fatality that has been buried or cremated
R	recovered and immune to reinfection

As in the standard SEIR model, our model includes susceptible, exposed, infected and recovered stages. To account for the particular dynamics of EVD, our model also includes a hospitalized stage and buried and unburied fatality stages.

Exposure

Individuals become exposed to EVD via contact with infectious individuals in the community (I) or with the bodies of the deceased (F_C_). Thus, for individuals i and j, if i and j are neighbors on the transmission network and X_j_(t) ∈ {I, F_C_} and X_i_(t) = S:


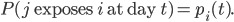


Under the baseline condition in which behavior change is not assumed, p_i_(t) = p_0_. When behavior change is considered, p_i_(t) decreases as the population's awareness increases. We selected the same exposure probability for infected individuals and unburied fatalities, due to the lack of conclusive studies on the comparative risk of transmission. We assumed no risk of transmission once the patient had been isolated in an ETU (H) in order to maximize the efficacy of ETU expansion. We will describe the effects of awareness on transmission in detail in later sections.

Infection and Isolation

All exposed individuals progress to the infectious stage following the incubation period. If individual i is exposed at day t, then


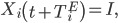


where T_i_^E^ is the incubation period for individual i, which is drawn from the incubation times distribution (Figure 5). We used an incubation time distribution that was fit to observed incubation times from prior EVD outbreaks and has been used in other modeling efforts 12.

Isolation of infected individuals is one of the key factors in EVD containment. We assume that two factors determine whether or not an infected individual will be hospitalized: (1) the individual's willingness to seek treatment for EVD and (2) the availability of such treatment. When there are shortages of ETU beds for those seeking treatment, individuals are selected randomly to fill the ETUs to capacity.

Recovery and Death

Once infected, individuals eventually either recover or die from EVD. Let Q_i_ define whether individual i will recover or die. Then,


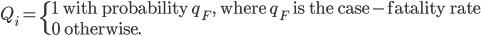


We assume that the case-fatality rate among hospitalized individuals is not different than that among infectious individuals in the community, though we note that hospitalized patients may have a slightly increased probability of survival 9

Both the length of time from symptom onset to recovery and from symptom onset to death are modeled using gamma distributions fit to data collected by WHO^9^, using method of moments estimators 13, and are plotted in Figure 5. For an individual i who becomes infected on day t:

If Q_i_ = 1,


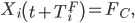


where T_i_^F^, the number of days between symptom onset and death for individual i, is drawn from the time until death distribution.

Otherwise, if Q_i_ = 0,


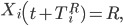


where T_i_^R^, the number of days between symptom onset and recovery for individual i, is drawn from the time until recovery distribution.

Recovered individuals (R) become immune to reinfection and can no longer transmit the disease. Unburied fatalities (F_C_) continue to transmit infection until safely buried or cremated (F_B_). We assume that patients who die in ETUs are safely and quickly buried, and pass immediately from the hospitalized (H) to the buried (F_B_) stage. Individuals who die outside of ETUs are assumed to be buried at most T^B^ days after death.

**Social Mobilization Model**

We overlay the EVD transmission and case isolation model with a model of social mobilization, in which social mobilization programs reach community members and increase their awareness of EVD. Community members then communicate about the disease, arriving at a consensus level of awareness. We assume that increased awareness decreases: (1) the delay between symptom onset and presentation at a treatment center, (2) the delay between death and burial and (3) the transmission probability.

Spread of Awareness via Social Mobilization

Let Y_i_(t) ∈ [0, 1] denote individual i's awareness of and willingness to seek treatment for EVD at time t. An individual with an awareness of 1 will seek treatment immediately; an individual with an awareness of 0 will not seek treatment. The initial level of awareness in the community is Y_0_. That is,


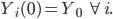


In simulations in which there is no social mobilization, awareness is fixed at Y_0_. In simulations in which social mobilization takes place, awareness increases over time. Social mobilization is modeled as follows. Every day each individual's awareness level is updated to the average awareness of the community, following a social mobilization effort described by the function f_M_. Thus,


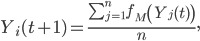


where n is the size of the population and where


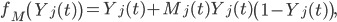


where M_j_(t) ∈ {0,1}. We assume that the social mobilization campaign begins on day T^M^. (In Lofa County, T^M^ = 42 32.) M_j_(t) = 1 if t ≥ T^M^ and j is reached by social mobilization (with probability η) and M_j_(t) = 0, otherwise. The increase in awareness is dependent on the size of the mobilization efforts (η) and incorporates initial resistance to mobilization efforts (Figure 6).

Effect of Awareness on EVD Transmission

The delays between symptom onset and treatment seeking and between death in the community and burial are determined probabilistically, according to the affected individual's awareness. As awareness increases, the probability of isolation in an ETU increases, as does the probability of being quickly and safely buried. Assuming that there is space available in the ETU and X_i_(t) = I, then:


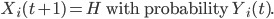


Similarly, if X_i_(t) = F_C_, then:


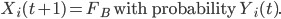


We also consider reductions in the transmission resulting from preventative measures taken within the community and within households, such as self-isolation and avoidance of public places 32 ^,^ 35. Each infected individual or fatality to which individual i is connected on the disease network has probability p_i_(t) of exposing i at time t, where p_i_(t) is defined as follows:


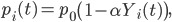


and where α ∈ [0, 1] is the maximum reduction in the per-contact exposure probability resulting from awareness of the disease.

**Study Design and Implementation**

Data

The model was fit to weekly EVD cases in Lofa County between the week ending June 16, 2014 and the week ending November 1, 2014, compiled by the US CDC 31, based on information provided by the Liberian Ministry of Health and Social Welfare (MoHSW) and local Lofa County health offices. According the Liberian MoHSW, 623 cases were reported, giving Lofa County the third highest cumulative incidence of the disease in Liberia 31. Point estimates of the number patients isolated in ETUs were also provided by the Liberian MoHSW 27. Since there have been many reports of shortages of ETU capacity 29 ^,^ 36, it was important to account for the capacity of the ETUs in order to gain an accurate picture of the outbreak dynamics. We estimated the capacity of the Lofa County ETUs on a given day as the maximum number of patients actually reported in ETUs prior to or on that day (Figure 3). By using the reported number of patients, instead of the reported number of beds, as an estimate of capacity, we accounted for the fact that patients in ETUs commonly shared beds or were placed on the floor. This assumption may have resulted in a slight underestimation of capacity, but the underestimation was not as severe as it would have been if we had assumed that ETU capacity was equal to the reported number of beds. We accessed a cleaned version of the reported ETU admissions on Github 37. We also considered reports from WHO and UNICEF describing the social mobilization effort 26 ^,^ 28 ^,^ 32. Based on these reports, we determined that social mobilization efforts began to change behaviors in mid-July, approximately 6 weeks (42 days) after the beginning of the outbreak.

**Estimated capacity of Lofa County ETUs from May 23 to October 23, 2014.**
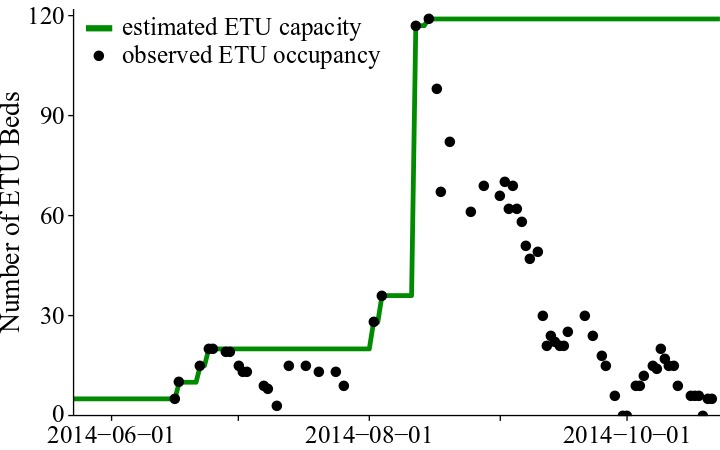
We estimated the capacity of the Lofa County ETUs on a given day as the maximum number of patients actually reported in ETUs prior to or on that day. We derived this estimate based on two assumptions: (1) the capacity of an ETU is greater than the number of beds in the ETU, because patients can share beds or be placed on the floor, and (2) ETUs were likely operating at or near capacity during the early stages of the outbreak.

Implementation

We used a small-world network 38 to represent the local clustering of individuals likely to be in close contact with the infected, e.g. family members or close friends. We selected 8 as the mean degree of the network, because the average size of a household in Lofa County is 5.5 people 39, and we assumed that, in addition to household members, most individuals would have several close friends or relatives. The rewiring probability was chosen to be 0.01. The entire Lofa County population of 276,863 39 was represented in the network. Transmission parameters were extracted from the literature and are listed in Table 1. The baseline per-contact exposure probability and the social mobilization parameters were fit to the weekly case counts and are listed in Table 2.

Since the reported cases of EVD represent only a fraction of the total number of cases 40, it was necessary to account for under-reporting. We assumed that cases that sought treatment or were safely buried were documented in the official case counts. Survivors who never sought treatment and fatalities that were not safely buried were not documented. Therefore, in our simulations, we fit to only cases that sought treatment or were safely buried, but modeled all cases. We postulated that as awareness increases, the number of unreported cases decreases, which is consistent with the observed changes in the number of EVD tests conducted in Lofa County from June through October 31.

We fit the model by performing a greedy search of the parameter space, with random restarts. One-thousand instances of the model were executed for each combination of parameters. All simulations were initialized with a single infected individual on May 23, 2014 26. The quality of the simulated fit was determined by comparing the median simulated weekly reported cases with the observed weekly reported cases. We used mean absolute error (MAE) as the fitting metric. Let m(w) be the median number of new reported cases predicted by the model during week w, and let M(w) be the actual number of new reported cases during week w according to the CDC data. Then the MAE is defined as follows:

\begin{equation*}\mbox{MAE} = \frac{1}{W_{\mbox{max}}} \sum_{w=1}^{W_{\mbox{max}}} \left|m(w) - M(w)\right|,\end{equation*}

where week 1 is the week ending June 16, 2014 (the first week included in the CDC data) and W_max_ is the last week fit to data.

**Table 1. Parameters extracted from the literature.** 

Parameter	Description	Value	Reference
T_i_^E^	days from exposure until symptom onset for individual i	discrete distribution derived from data	12
q_F_	case-fatality rate	0.708	9
T_i_^R^	days from symptom onset until recovery for individual i	T_i_^R^ ~Gamma(k = 6.37, θ = 2.58)	9
T_i_^F^	days from symptom onset until death for individual i	T_i_^F^ ~Gamma(k = 1.22, θ = 6.17)	9
T^B^	days between death and burial	2	12 ^,^ 29
T^M^	days until social mobilization begins to affect awareness	42	32

**Table 2. Ranges of parameters used to fit the model to data:** 

Parameter	Description	Range with Behavior Change	Range without Behavior Change
p_0_	baseline per-contact probability of exposure	0.180, 0.185, ... ,0.340	0.180, 0.185, ... , 0.340
α	maximum reduction of p_0_ resulting from awareness	0.0, 0.1, ... , 0.8	0.0
Y_0_	initial level of awareness in the community	0.10, 0.15, ... , 0.30	0.10, 0.15, ..., 0.30
η	daily probability of being reached by social mobilization	0.00, 0.01, ... , 0.10	0.0

Intervention Scenarios

There was a remarkable decrease in the rate of EVD transmission from the first months of the Lofa County outbreak to the later months. Starting in mid-August, the rate of transmission slowed substantially (Figure 1). We designed a set of three scenarios to understand the role of social mobilization efforts in helping to contain the spread of EVD. In all scenarios, ETUs were expanded to the levels actually observed in Lofa County. Behavioral change in the population was considered in only one of the scenarios.

*Scenario 1: Extrapolation from Early Epidemic Data assuming Increased ETU Capacity and No Behavior Change*

Scenario 1 parameters were selected to minimize the MAE for weeks ending during the months of June and July (W_max_ = 7). The behavior change parameters, α and η, were fixed to 0, indicating no change in population behavior. Since this scenario fits to early epidemic data (prior to the change in behaviors), it allows us to predict the course of the outbreak if behavior change had never taken place. The results of this scenario should be interpreted with caution, however, as they are derived from parameters fit to a relatively small amount of data.

*Scenario 2: All Data Fit assuming Increased ETU* *Capacity and No Behavior Change*

Scenario 2 parameters were selected to model the outbreak through the end of October by minimizing the MAE for the months June through October (W_max_= 21). This scenario tested if model parameters could be found that would accurately model the course of the outbreak, assuming no behavior change in the population.

*Scenario 3: All Data Fit assuming Increased ETU Capacity and Behavior Change*

In Scenario 3, we added effects for social mobilization efforts (α ≥ 0 and η > 0). We selected parameters that minimized the MAE for the months June through October (W_max_= 21). Scenario 3 tested if a good fit to the observed weekly cases could be obtained, if behavioral changes were taken into account.

## Results

The results of the simulations show that fitting to early epidemic data without considering behavioral change results in a significant overestimation of the size of the outbreak; modeling the entire outbreak without considering behavioral change provides a poor overall fit; and the introduction of behavioral changes to the model provides a good fit to the observed data (Figure 4).

**Best fit models illustrate that changes in human behavior must be considered in order to adequately fit the observed cumulative cases of EVD in Lofa County, Liberia.**
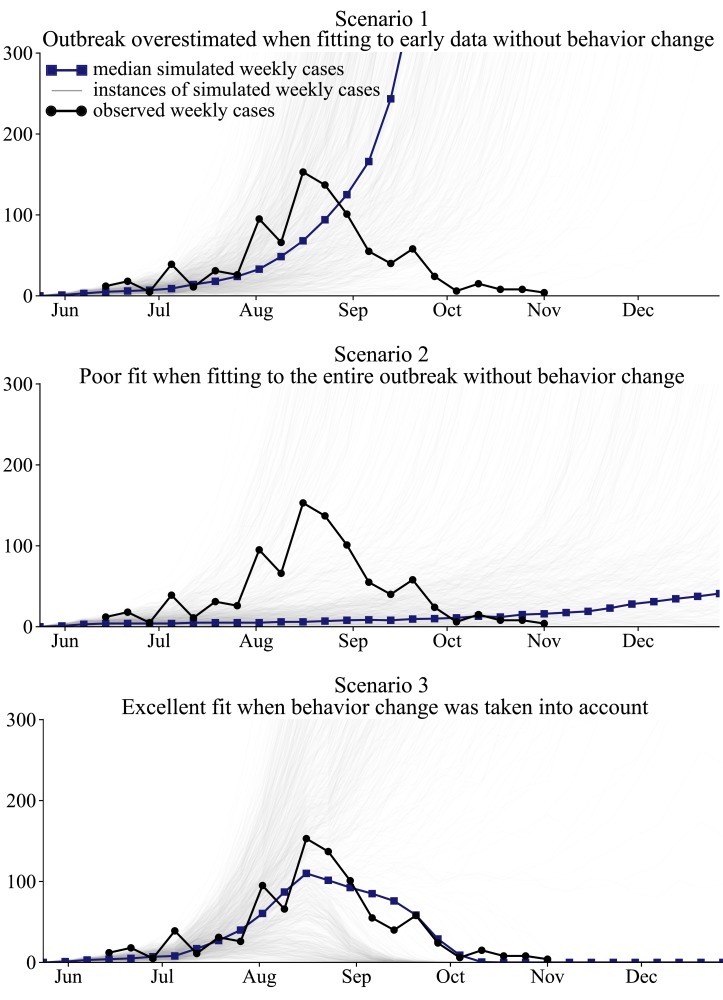
In Scenario 1 and Scenario 2, the capacity of the Lofa County ETUs was increased, as actually occurred, but no behavior change took place in the population. Scenario 1 fit the weekly case counts in June and July and extrapolated the trend to future months. Scenario 1 substantially overestimated the size of the outbreak, predicting 13,411 or more reported cases by November 1 in 50% of simulations. Scenario 2 fit the weekly cases for June through October. A poor fit to the observed cases was obtained. Scenario 3 took into account gradually increasing awareness of EVD in the community, in addition to the expansion of the Lofa County ETUs, achieving an excellent fit to the observed weekly cases.

For Scenario 1, we achieved a good model fit to the observed cumulative cases from June through July, with a MAE of 9.9 cases during that period (Figure 4). However, the simulations predicted an increasingly worsening outbreak in September and October, instead of the improvements that were actually seen (Figure 4). Of 1000 simulations, 50% predicted 13,411 or more reported cases by November 1. In 65% of simulations, 5000 or more reported cases were predicted by November 1. In reality, no new cases of EVD were reported in Lofa County in the 3 weeks ending November 22 36, indicating the virtual end of the outbreak. We found that only 16% of 1000 simulations anticipated the end of the outbreak by November 22, assuming that no additional capacity was added to the Lofa County ETUs after November 1.

Scenario 2 simulations were designed to test if the Lofa County EVD outbreak could be adequately modeled without accounting for behavioral changes. Simulations provided a poor fit to the observed weekly cases (Figure 4). The weekly cases were underestimated from June through September and overestimated in late October. In October, the simulated outbreaks were worsening, rather than improving as actually observed. The outbreak ended by November 22 in 42% of follow-up simulations.

The Scenario 3 simulations, which considered behavior change resulting from increased awareness, provided the best fit to the observed weekly cases (Figure 4). The median number of new cases reported during the 21 weeks ending on November 1 in Scenario 3 simulations was 835, which is close to the observed value in the CDC data, 912 ^23^. (Note that 912 is the sum of the weekly new cases. The number of cumulative cases, 623, is lower due to reclassification.) The outbreak ended by November 22 in 51% of simulations. There was substantial variability in Scenario 3 simulations. The reduction in community transmission resulting from awareness was only sufficient to bring the reproduction number (the expected number of secondary cases per infected individual) below 1 if there was sufficient capacity in the ETUs to isolate a large proportion of cases. In simulations in which the outbreak grew rapidly in June and July, the ETUs were filled to capacity in later months, and too few cases were isolated to slow the exponential growth of the outbreak. The best fit parameters for each scenario are listed in Table S1.

## Discussion

Our findings suggest that behavioral change in the population of Lofa County was instrumental in bringing the outbreak under control. Under the counter-factual scenario in which we assumed that no behavioral change took place, the increase in capacity of the Lofa County ETUs in mid-August was insufficient to bring the outbreak under control in most of our simulations. Instead, the outbreak continued to spread, infecting over 13,000 people in most simulations by November 1, 2014. We therefore believe that behavioral change resulting from a successful social mobilization campaign may have averted hundreds, if not thousands, of EVD cases in Lofa County.

Based on our case study of Lofa County, we believe that behavioral change is playing a significant role in slowing the spread of disease and that the observed dynamics of the outbreak cannot be fully explained by the increase in treatment capacity alone. It remains to be seen whether incorporating behavioral change into predictive models will be feasible, given the lack of availability of high-quality data on human behaviors. Nevertheless, models that do not take into account behavioral effects will likely produce overly pessimistic predictions, especially when extrapolating from early epidemic data, as did our Scenario 1 model. During the initial growth stage of the outbreak, there was low awareness of prevention methods, resulting in a higher per-contact transmission probability than was observed at later points in the outbreak, when available preventative measures were already in place.

Our model results for Lofa County support continued focus on social mobilization as a means to combat the spread of EVD. Education and awareness programs cannot be a substitute for traditional interventions, but they can augment these efforts by preventing transmission within the community and ensuring the cases and fatalities are reported promptly. As response efforts are implemented to increase the capability to isolate and safely bury cases, social mobilization efforts can help to make these efforts as effective as possible.

## Competing Interests

The authors have declared that no competing interests exist.

## Supplementary Material

**Time until event distributions used in the model.**
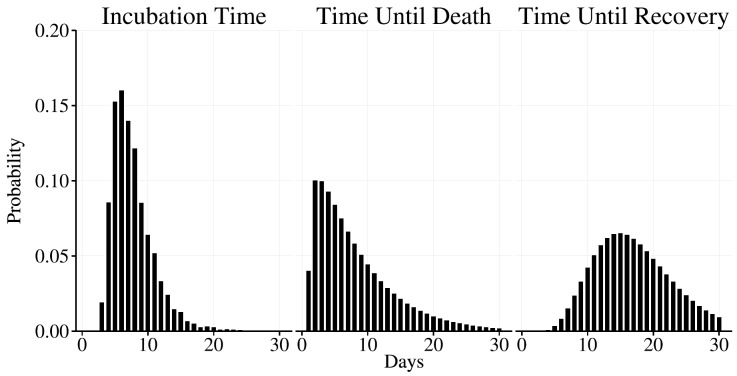
The incubation time (T_i_^E^) was drawn from the incubation time distribution. For fatalities, T_i_^F^ was drawn from the time until death distribution. For survivors, T_i_^R^ was drawn from the time until recovery distribution.

**Social mobilization efforts increase awareness gradually.**
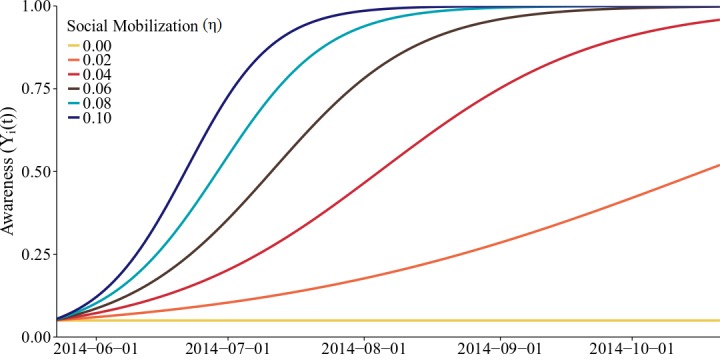
Change in awareness over time is shown for values of social mobilization ranging from 0.0 to 0.1.

**Table S1. Parameters of best fit models.** 

Scenario	p_0_	α	Y_0_	T^M^	η
1: Extrapolation from early epidemic data assuming an increase in ETU capacity and no behavior change	0.310	0.0	0.15	0	0.00
2: All data fit assuming an increase in ETU capacity and no behavior change	0.335	0.0	0.30	0	0.00
3: All data fit assuming an increase in ETU capacity and behavior change	0.305	0.7	0.10	42	0.06
